# Comprehensive Analysis of PANoptosis-Related Gene Signature of Ulcerative Colitis

**DOI:** 10.3390/ijms25010348

**Published:** 2023-12-26

**Authors:** Jun-Meng Wang, Jiao Yang, Wan-Yu Xia, Yue-Mei Wang, Yuan-Bing Zhu, Qin Huang, Tong Feng, Lu-Shuang Xie, Si-Hui Li, Shu-Qing Liu, Shu-Guang Yu, Qiao-Feng Wu

**Affiliations:** 1 Acupuncture and Moxibustion School, Chengdu University of Traditional Chinese Medicine, Chengdu 611137, China; 2 School of Basic Medicine, Chengdu University of Traditional Chinese Medicine, Chengdu 611137, China; 3 Acupuncture & Chronobiology Key Laboratory of Sichuan Province, Chengdu 611137, China; 4 Key Laboratory of Acupuncture for Senile Disease, Chengdu University of TCM, Ministry of Education, Chengdu 611137, China

**Keywords:** PANoptosis, colitis, integrated analysis, gene signatures, ceRNA network

## Abstract

Accumulating evidence shows that the abnormal increase in the mortality of intestinal epithelial cells (IECs) caused by apoptosis, pyroptosis, and necroptosis is closely related to the function of mucous membrane immunity and barrier function in patients with ulcerative colitis (UC). As a procedural death path that integrates the above-mentioned many deaths, the role of PANoptosis in UC has not been clarified. This study aims to explore the characterization of PANoptosis patterns and determine the potential biomarkers and therapeutic targets. We constructed a PANoptosis gene set and revealed significant activation of PANoptosis in UC patients based on multiple transcriptome profiles of intestinal mucosal biopsies from the GEO database. Comprehensive bioinformatics analysis revealed five key genes (ZBP1, AIM2, CASP1/8, IRF1) of PANoptosome with good diagnostic value and were highly correlated with an increase in pro-inflammatory immune cells and factors. In addition, we established a reliable ceRNA regulatory network of PANoptosis and predicted three potential small-molecule drugs sharing calcium channel blockers that were identified, among which flunarizine exhibited the highest correlation with a high binding affinity to the targets. Finally, we used the DSS-induced colitis model to validate our findings. This study identifies key genes of PANoptosis associated with UC development and hypothesizes that IRF1 as a TF promotes PANoptosome multicomponent expression, activates PANoptosis, and then induces IECs excessive death.

## 1. Introduction

Extensive epithelial erosion resulting from abnormally increased intestinal epithelial cells (IECs) mortality has become a common feature of several intestinal diseases, such as inflammatory bowel disease and infectious colitis [1]. Excessive death of IECs will not only cause intestinal barrier dysfunction and bacterial translocation [2], but will also cause secondary inflammation and aggravate mucosal epithelial damage [3]. Current biologics play some role in treatment, but many non-responding patients exhibit unimproved mucosal manifestations, which may be related to complex redundant or co-activation mechanisms between different types of epithelial cell death [4]. 

As a controlled mode of cell death, programmed cell death (PCD) plays an essential role in host defense against pathogens and maintaining homeostasis [5]. However, excess activation of PCD pathways has proven to be detrimental and can drive disease [6]. In addition to apoptosis [7], the most classic form of PCD, other non-apoptotic forms of PCD, including pyroptosis [7,8] and necroptosis [9], have also been reported to be closely related to the colonic innate immune response in UC. These different forms of cell death molecular mechanisms are believed to not be independent [5,10], but do have crosstalk [11,12]. 

With the deepening understanding of the interaction of different death types, PANoptosis, a new broad programmed death pathway, has been proposed [13]. PANoptosis is defined as a cell death pathway regulated by the PANoptosis complex (PANoptosome), which has the essential characteristics of various cell death modes such as pyroptosis, apoptosis, and necroptosis [14], but cannot be interpreted by just one of them. As a key regulator of PANoptosis signaling, the PANoptosome is composed of sensors, adaptors, and effectors. Upstream sensors (ZBP1, AIM2, etc.) regulate these PCD pathways cascade, which assemble multi-component complexes (adaptors: ASC, FADD, etc.) as platforms for downstream molecule (effectors: RIRK1/3, CASP1/6/8, etc.) activation [15]. While various pathogenic and sterile cell injuries can lead to the formation of PANoptosome, the components of PANoptosome, especially the apical sensor that induces PANoptosome assembly, exhibit specificity [16]. So far, four distinct characterizations of PANoptosomes have been identified, namely Z-DNA-binding protein 1 (ZBP1), AIM2, receptor-interacting serine/threonine-protein kinase 1 (RIPK1), and NLR family pyrin domain-containing 12 (NLRP12) PANoptosomes [17,18]. These PANoptosomes, as crucial components of the innate immune response, are associated with infection, inflammatory diseases, and cancer [19]. However, the role of PANoptosis in UC and the composition of PANoptosome in the UC colon are largely unknown. Given that manipulating cell death and inflammation for therapeutic intervention is a subtle process highly specific to the context of related diseases [6,20], understanding the molecular mechanisms of PANoptosis and the components of PANoptosome in UC is crucial and may pave the way for the development of promising new strategies.

In this study, we identified pivotal PANoptosis-related molecular mechanisms and their relationship with disease severity and biologics in UC pathogenesis. In addition, we conducted immune infiltration, CeRNA network, and animal model validation to describe the impact of PANoptosis in UC. These understandings of the relationship between UC and PANoptosis is expected to contribute to the comprehension of mechanisms of IECs “Death Landscape” and find a new therapy for inducing and maintaining mucosa healing.

## 2. Results

### 2.1. GSEA Revealed the Innate Immune-Related Biological Processes between Healthy Individuals and UC Patients

**Figure 1:**
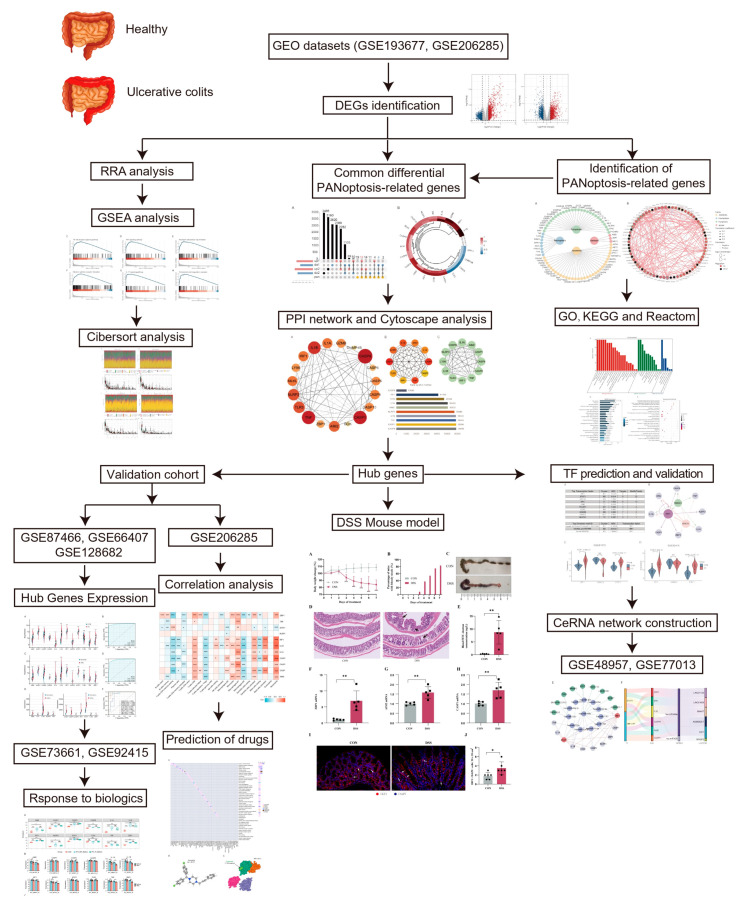
Flowchart of the study. The datasets GSE193677 and GSE206285 were used as training sets to obtain differentially expressed genes (DEGs) between healthy individuals and patients with ulcerative colitis (UC). RRA analysis combined with GSEA was used to identify enriched pathways of DEGs. The intersection of DEGs and PANoptosis genes resulted in the identification of differential PANoptosis-associated genes (DPEGs). CytoHubba and MCODE gene network analysis tools were used to identify hub genes of PANoptosis in a UC colon. Immunoinfiltration analysis and correlation analysis were conducted to determine the relationship between hub genes and immune cells. To validate the importance of hub genes in UC, the expression trends and responsiveness to biological agents of hub genes were verified in five external validation sets (GSE87466, GSE66407, GSE128682, GSE73661, and GSE92415). Subsequently, key transcription factors regulating PANoptosis were predicted, and a CeRNA regulation network of PANoptosis was constructed. Finally, the expression of key hub genes and transcription factors was validated in a classic UC animal model to support our analysis results.

**Figure 2:**
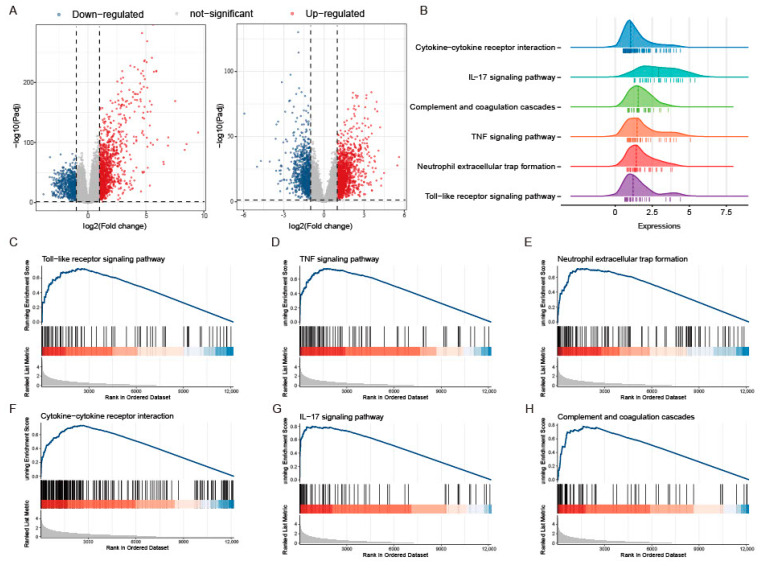
GSEA analysis of differential genes between UC and CON groups in two training sets. (**A**) Volcano plot representation of differential gene expression in GSE193677 and GSE20285. The red points represent up regulated genes and the blue points represent down regulated genes. (**B**) Ridgeline plots show immune-related pathways of GSEA based on the RRA-sorted rank gene. (**C**−**H**) Gene set enriched in the Toll-like receptor pathway (NES = 2.072, FDR = 5.82 × 10^−6^), gene set enriched in TNF signaling (NES = 2.236, FDR = 4.77 × 10^−9^), gene set enriched in the neutrophil extracellular traps formation (NES = 2.061, FDR = 3.82 × 10^−7^), gene set enriched in cytokine-cytokine receptor interaction (NES = 2.231,FDR = 4.13 × 10^−9^), gene set enriched in IL-17 signaling (NES = 2.665, FDR = 4.13 × 10^−9^), gene set enriched in Chemokine signaling pathway (NES = 1.989, FDR = 1.88 × 10^−7^). NES: normalized enrichment score.

The study workflow is presented in Figure 1. The sets GSE193677 and GSE206285 were normalized, and the gene expression with biological significance was obtained. In set GSE193677, there were 1916 genes differentially expressed between 293 UC samples and 461 healthy control samples, including 1240 upregulated genes and 676 downregulated genes (Figure 2A and Supplementary Table S2). For another independent set GSE206285, which contained 550 UC samples and 18 healthy control samples, there were 1311 upregulated genes and 1073 downregulated genes identified (Figure 2A and Supplementary Table S3). To remove the noise and bias between the data sets as much as possible and improve the stability and consistency of the results, we integrated the two training sets through RRA analysis to determine the differential genes with stable changes. After the genes were sorted according to RRA-Score, GSEA analysis was performed. As previously found, GSEA showed that the pathways related to innate immunity were significantly enriched (Figure 2B), including the toll-like receptor pathway, TNF signaling, neutrophil extracellular traps formation, cytokine-cytokine receptor interaction, IL-17 signaling, and chemokine signaling pathway (Figure 2C). 

### 2.2. Colonic PANoptosis Signaling Is Significantly Activated in UC Patients

**Figure 3:**
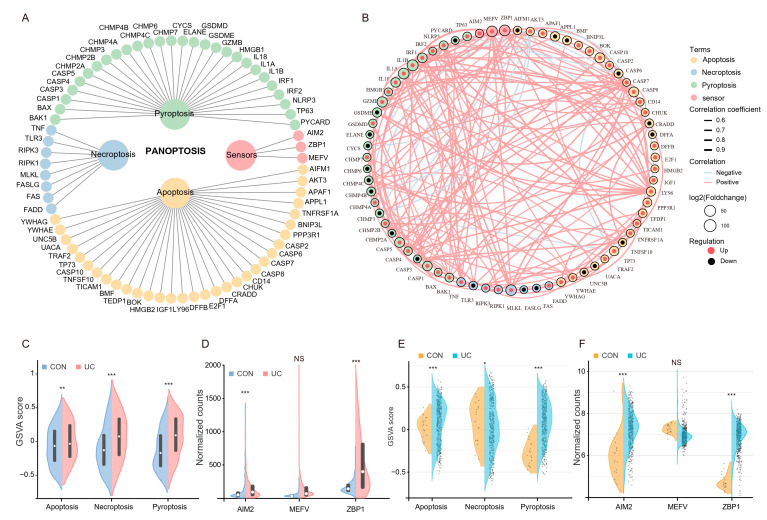
Colonic PANoptosis signaling is significantly activated in UC patients. (**A**) The PANoptosis gene list contains 71 genes, of which 28 are from pyroptosis, 32 are from apoptosis, 8 are from necroptosis, and 3 sensors reported to induce PANoptosis. (**B**) An interaction network of PANoptosis genes. (**C**) The expression patterns of PANoptosis gene sets between CON an UC group in GSE193677. (**D**) The expression patterns of PANoptosis gene sets between CON an UC group in GSE206285. (**E**) Differential expression of sensors that induce PANoptosis in GSE193677. (**F**) Differential expression of sensors that induce PANoptosis in GSE206285. * FDR < 0.05, ** FDR < 0.01, *** FDR < 0.001, NS, no significance.

The PANoptosis gene list contains 71 genes, of which 28 are from pyroptosis, 32 are from apoptosis, 8 are from necroptosis, and 3 sensors were reported to induce PANoptosis (Figure 3A). Among them, 51 genes (71.8%) were upregulated (log2FC > 0), while 20 genes (28.2%) were downregulated (log2FC < 0). Pearson correlation analysis of gene expression showed that most of the PANoptosis genes were positively correlated with each other (Figure 3B). We then scored the PANoptosis sub-pathway for each sample in the two training sets by GSVA analysis to determine whether it was altered between healthy controls and UC patients. The results showed that the GSVA scores of apoptosis, pyroptosis, and necroptosis in UC patients were significantly higher than those in the control group (Figure 3C,D); meanwhile, in addition to MEFV, the expression levels of ZBP1 and AIM2, which were reported to induce PANoptosis, expressed in UC were also significantly increased (Figure 3E,F). The above results suggest that PANoptosis signaling may be activated considerably in UC patients.

### 2.3. Screening and Functional Enrichment Analysis of Differential PANoptosis Genes

**Figure 4:**
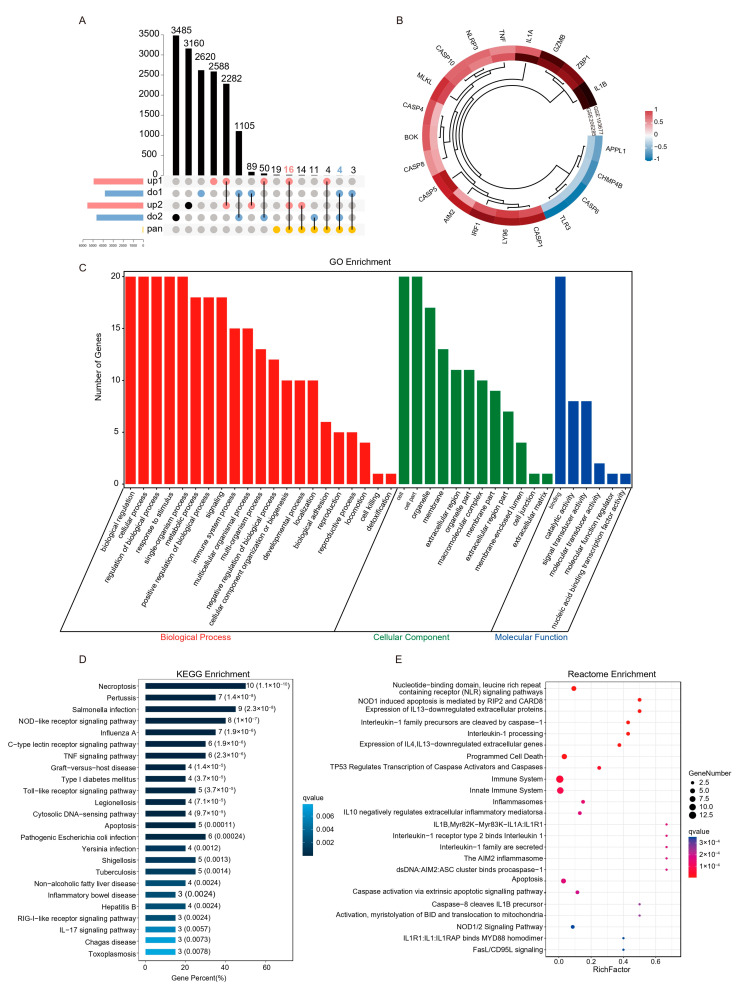
Screening and functional enrichment analysis of differential PANoptosis genes. (**A**) The upset plot shows the overlap between PANoptosis gene list and the same trend genes in the two training sets. (**B**) Heatmap representation of log2(Foldchange) of DEPGs in the two training sets. (**C**) GO functional enrichment analysis, including BP, CC, and MF, revealed the underlying functions of DEPGs. (**D**) KEGG pathway enrichment revealed the top 25 pathways enriched in DEPGs. (**E**) Reactome pathway analysis of the DEPGs.

To obtain reliable differential PANoptosis gene (DEPGs), we screened the intersection of differential genes with the same trend and PANoptosis genes list in the two data sets based on the standard of |Fold Change| ≥ 1.5 (Figure 4A). A total of 20 DEPGs were screened, 17 were up-regulated, and 3 were down-regulated (Figure 4B). Furthermore, GO, KEGG, and Reactome functional enrichment analyses were performed to determine the biological features of these DEPGs. GO functional enrichment analysis revealed 960 terms (FDR < 0.05) across BP, CC, and MF categories. DEPGs were markedly enriched in biological regulation, cellular process and response to stimulus in the BP category. In the CC category, genes were mainly enriched in the organelle and membrane. Enriched MF terms included binding, signal transducer activity, and catalytic activity (Figure 4C). KEGG pathway analysis revealed genes were mainly enriched in Necroptosis, TNF signaling pathway, and Apoptosis signaling pathways (Figure 4D). Reactome analysis revealed that genes were mainly enriched in NOD1 induced apoptosis, Interleukin-1 processing, and programmed cell death terms (Figure 4E). These results indicated that PANoptosis-related cell death and pro-inflammatory pathways were enriched in UC patients.

### 2.4. The PPI Network Analysis Indicated That the Hub Gene Mainly Comprises PANoptosis Sensors and Effectors

**Figure 5:**
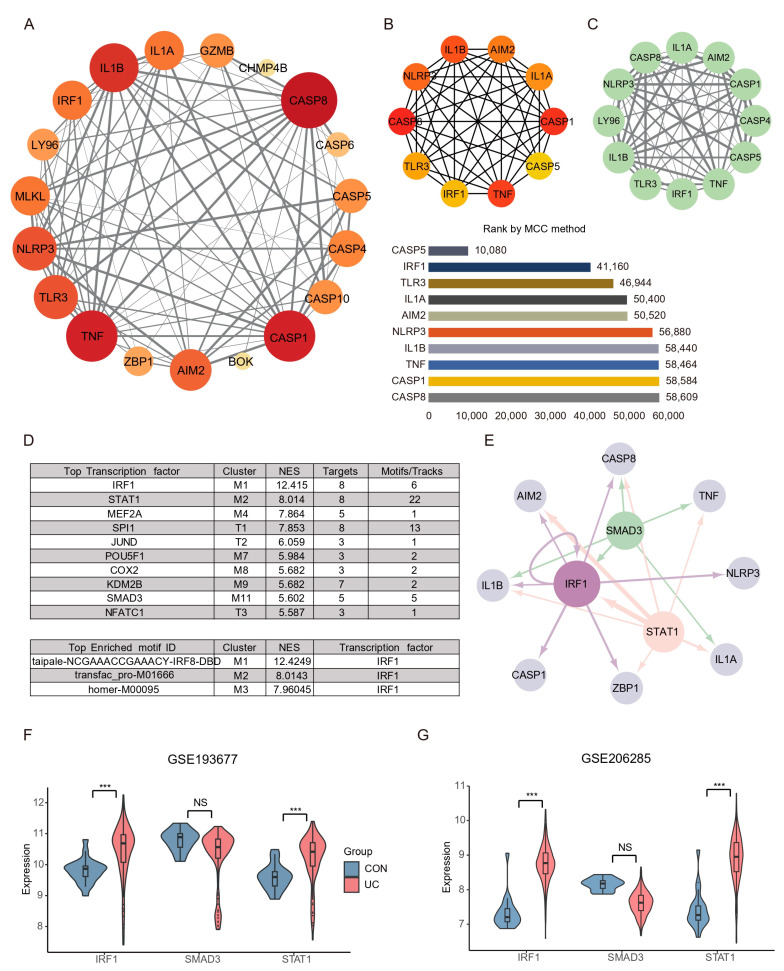
PPI network construction and key TFs identification PANoptosis. (**A**) Representative protein–protein interaction network of DEPGs. The color and size of the dots represent the connection frequency, and the thickness of the lines represent the combined score. (**B**) cytoHubba was used to identify hub nodes in the network based MCC method. (**C**) MCODE identify a sub-network cluster 1 with 12 nodes. (**D**) iRegulon predicted the top ten transcription factors and the top three motifs. (**E**) Hub genes and transcription factors network based on NES (≥5), targets (≥5), and motif (≥5) screening. Light gray nodes represent hub genes, and other colors represent transcription factors. The size of nodes means NES scores and the thickness of the connection represents the number of motifs predicted by transcription factors and hub genes. (**F**) TFs expression analysis in GSE87466. (**G**) TFs expression analysis in GSE92415. *** FDR < 0.001, NS, no significance.

An interaction network between the 20 DEPGs was constructed using the STRING (v11.5) database (Figure 5A). The interaction network comprised 20 nodes and 89 edges, visualized using the Cytoscape software. The cytoHubba plugin was used to identify hub nodes and obtained 10 hub nodes with MCC methods (Figure 5B). Next, The MCODE plugin was used to identify gene cluster modules and obtained one Cluster 1 that had the high score (score: 10.364; Figure 5C). A complex gene regulatory network regulates gene expression, and TFs are the core factors in the gene regulatory network, affecting development, homeostasis, and pathogenesis through interactions with target genes. Therefore, we predicted the TF of the hub gene by iRegulon plugin. The results show that among the top ten TFs with predicted scores NES, only STAT1, IRF1, and SMAD3 have more than five target genes and motifs, and the top three motifs with predicted scores all point to IRF1 (Figure 5D). Interestingly, IRF1 is not only a hub gene, but also acts as a transcription factor of AIM2, CASP8, IL1B, CASP1, ZBP1, and itself (self-feedback loop), suggesting that IRF1 may play an essential role in the PANoptosis process of UC colon (Figure 5E). Subsequently, we verified the expression of key TFs through the validation sets, and the results showed that STAT1 and IRF1 were significantly increased in the UC group, while SMAD3 had no difference (Figure 5F,G).

### 2.5. Patients with Active UC and Inflammatory Tissues of the Colon Exhibit High Expression of Hub Genes and TFs of PANoptosis

**Figure 6:**
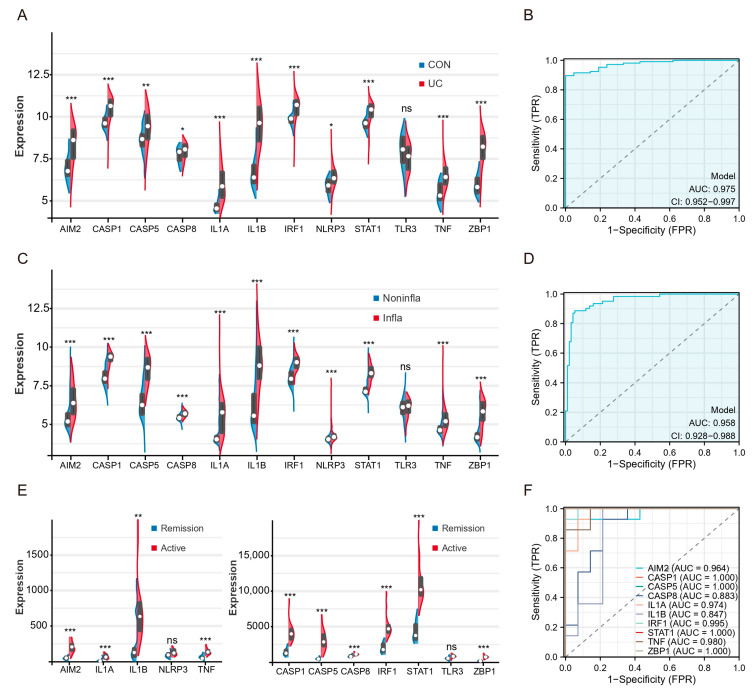
Patients with active UC and inflammatory tissues of the colon exhibit high expression of hub genes and TFs of PANoptosis. (**A**) Split violin plot revealing the expression differences in hubs between UC patients and healthy controls in the GSE87466. (**B**) ROC curves based on hubs combination in the GSE87466. (**C**) Split violin plot revealing the expression differences in hubs between inflammatory tissues and non-inflammatory tissues in the GSE66407. (**D**) ROC curves based on hubs combination in the GSE66407. (**E**) Split violin plot revealing the expression differences in hubs active UC and remission patients in the GSE128682. (**F**) ROC curves based on single hub in the GSE128682. * *p* < 0.05, ** *p* < 0.01, *** *p* < 0.001, NS, no significance.

To validate the expression levels of hubs and TFs, we used three external validation datasets (GSE87466, GSE66407, GSE128682). The expression levels of hubs in the colon tissue of UC patients were significantly higher compared to healthy controls, except for TLR3 (Figure 6A). ROC analysis showed that the AUCs of multigene combination of all differential hubs were >0.95 (Figure 6B). The levels of these genes were also found to be higher in inflammatory tissues compared to non-inflammatory tissues in the GSE66407 (Figure 6C). The AUCs of the multigene combination of 11 genes were >0.95 (Figure 6D). Then, we analyzed the levels of these genes between active UC and remission patients in the GSE128682. All genes except TLR3 and NLRP3 were upregulated in active UC patients’ diseased colonic tissues (Figure 6E). Since the sample size in this dataset does not meet the statistical requirements of several times the genes of the independent variable, we analyze the predictive performance of each differential hub for active UC separately. ROC analysis based on a single gene showed that the AUC of 8 hub genes and 2 TFs exceeded 0.85.

### 2.6. Hub Genes and TFs of PANoptosis Are Associated with UC Patients’ Response to Biologics

**Figure 7:**
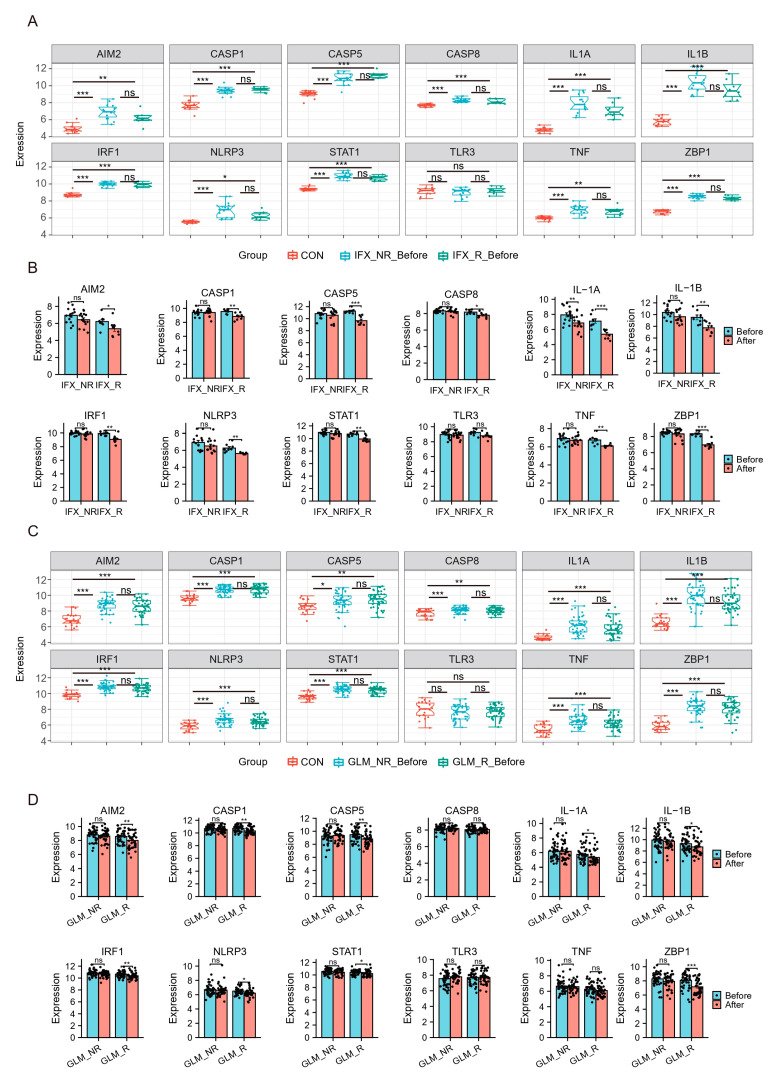
Hub genes and TFs of PANoptosis are associated with UC patients’ response to biologics. (**A**,**B**) The relative expression levels of hubs in the colonic mucosa of healthy controls, UC patients in responding and non-responding groups before and after IFX therapy in GSE73661. (**C**,**D**) The relative expression levels of hubs in the colonic mucosal of healthy controls, UC patients in responding and non-responding groups before and after GLM treatment in GSE92415. IFX, infliximab; GLM, golimumab. * *p* < 0.05, ** *p* < 0.01, *** *p* < 0.001, ns, no significance.

Recently, biologics such as TNF-a inhibitors infliximab (IFX) and golimumab (GLM) have been approved for treating ulcerative colitis and proposed as first-line agents. Still, some patients may not respond to biologics or respond poorly. The effects of biologics on hubs were explored using the GSE73661 and GSE92415. GSE73661 contains expression profiles of biopsy samples from UC patients treated with IFX. Before IFX treatment, hubs in UC patients except TLR3 were significantly elevated compared to healthy controls. There was no difference in Hubs and TFs expression levels in the non-response group (IFX_NR_Before) and the response group (IFX_R_Before) (Figure 7A). After IFX treatment, the expression of hubs except TLR3 was significantly reduced in the IFX clinical response group (IFX_R_After) (Figure 7B).

We also analyzed the GSE92415, which contains expression profiles of biopsy samples from UC patients treated with GLM. Before GLM treatment, the expression of hubs except TLR3 was higher in patients with active UC (Figure 7C). After GLM treatment, the expression of hubs except CASP8, TLR3, and TNF was decreased in the clinical response group (Figure 7E). These results indicated that IFX and GLM responders might improve impaired colonic mucosal in UC patients by regulating the PANoptosis signaling.

### 2.7. PANoptosis Is Associated with Increased Immune Cells and Proinflammatory Factors in the Colonic Mucosa of UC Patients

**Figure 8:**
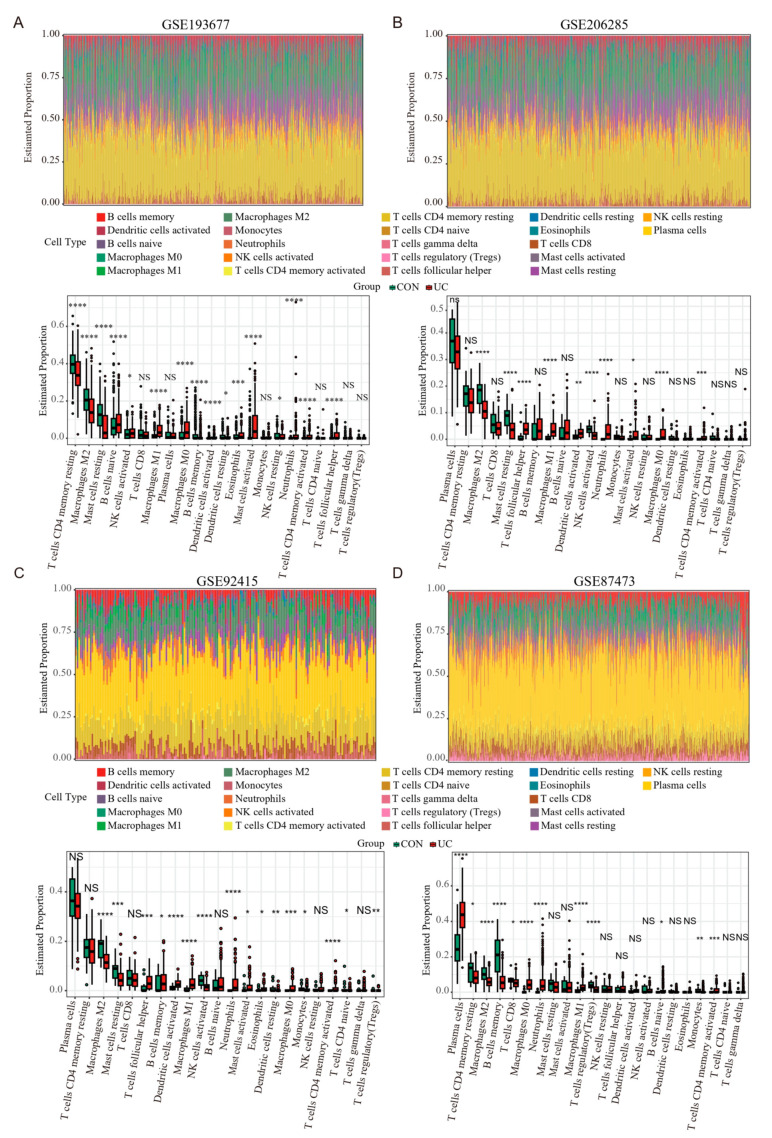
Estimation of infiltrating immune cell types in the 2 training and 2 validation GEO sets via CIBERSORT. (**A**–**D**) Barplots show the relative composition of 22 immune cell subsets in four sets, and the boxplots show that the difference in the proportion of immune cells between the CON and UC groups. Data were assessed via the method of Benjamini and Hochberg (BH). * adj. *p*-value < 0.05, ** adj. *p*-value < 0.01, *** adj. *p*-value < 0.001, **** adj. *p*-value < 0.0001, NS, no significance.

**Figure 9:**
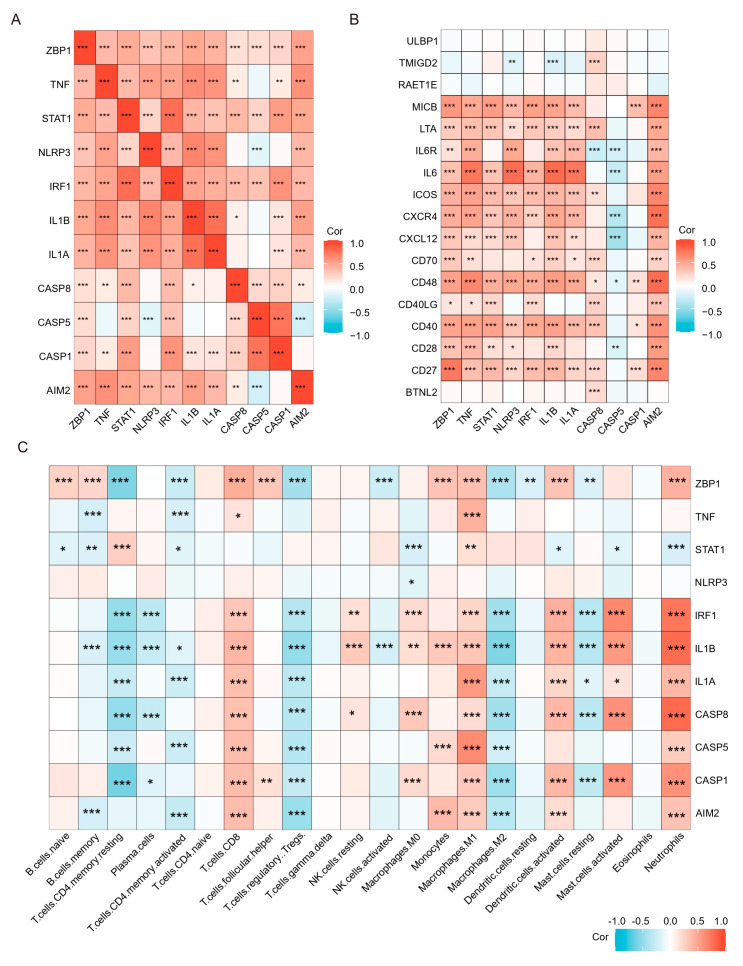
Correlation analysis of hub genes and TFs with immune cells and immunostimulatory factors. (**A**) Correlation analysis of the hub genes and TFs. (**B**) Correlation analysis of hub genes and TFs with immunostimulatory factors. (**C**) Correlation analysis of hub genes and TFs with 22 immune cell types. * *p* < 0.05, ** *p* < 0.01, *** *p* < 0.001.

Previous results have shown that PANoptosis signaling is significantly activated in inflammatory colonic tissue; we analyzed the immune landscape of the two training and two validation sets using CIBERSORT. The abundance of 22 immune cell types is shown using barplots (Figure 8A–D). The results revealed that the UC tissue was infiltrated by a higher fraction of Neutrophils, CD4 memory activated T cell, and macrophages (M0 and M1) and a lower fraction of M2 macrophages in all sets (FDR < 0.05), whereas other immune cells exhibited heterogeneity (Figure 8A–D). We then performed a correlation analysis on the expression data from the GSE206285 validation cohort. Except for CASP5, a positive correlation was observed between the hub genes and TFs (Figure 9A). In addition, correlation analysis also found that hub genes and TF, except CASP5, were positively correlated with most pro-inflammatory factors (Figure 9B). We further explored the relationship between hub genes and immune cells, and most of the hub genes and TFs were positively correlated with pro-inflammatory cells, such as neutrophils, M1 macrophages, activated mast cells, and activated DCs, and negatively correlated with anti-inflammatory cells, such as M2 macrophages (Figure 9C). These analyses confirmed that the hub genes and TFs of the PANoptosis pathway are closely related to the regulation of immune cell infiltration and immune regulatory factors, and their mechanism of action deserves further study.

### 2.8. Prediction of Potential Drugs Targeting PANoptosis Signaling in UC Patients

**Figure 10:**
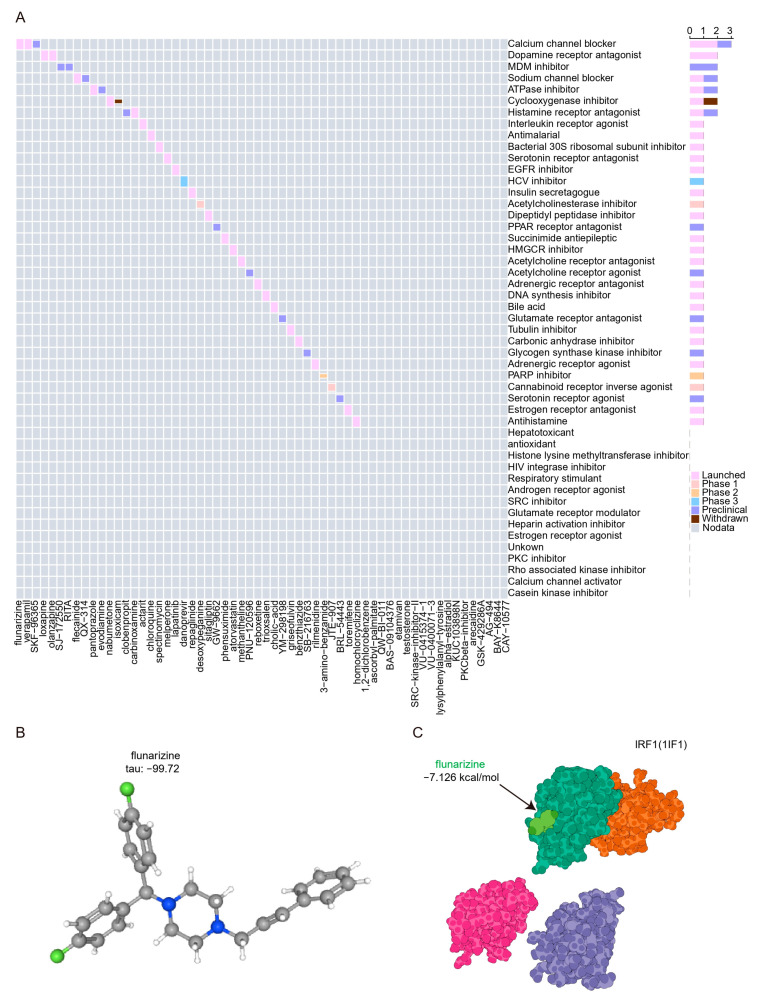
Prediction of potential drugs targeting PANoptosis signaling. (**A**) CMap analysis showed the mechanism of action based on small-molecule compounds. (**B**) 3D structures of flunarizine from PubChem open chemical database. The gray, blue, and green spheres represent carbon atoms, nitrogen atoms, and hydrogen atoms, respectively. (**C**) Molecular docking pattern of flunarizine complexed with IRF1. The light green and dark green colors represent the crystal structures of flunarizine and the binding of IRF1, respectively.

We submitted the 11 hubs and genes to the CMap database to screen for promising small molecule compounds that could be used for UC management. Based on the CMap connectivity score (tau), we screened the top 60 credible small molecule compounds (tau < −95) with reverse perturbations to the hub genes (Supplementary Table S4). The results show that the calcium channel blocker signal contains the most related compounds and has entered the clinical trial launch stage (Figure 10A). The structure of the compound flunarizine, which scored the lowest tau, was retrieved from the PubChem database and displayed in Figure 10B. Molecular docking is an important method for structure-based drug design and screening by finding the optimal conformation of small molecule compounds and target molecules for interaction. In this study, we downloaded high-resolution structural data (3 Å) from the RCSB Protein Data Bank for IRF1 (PDB ID: 1IF1), a key hub and transcription factor for PANoptosis signaling, to dock with flunarizine. The docking scores were less than −7 kcal/mol, suggesting a high binding affinity of flunarizine with the IRF1. The binding poses and sites are shown in Figure 10C, where the light green color represents the compounds.

### 2.9. Construction of Reliable ceRNA Regulatory Network for PANoptosis Signaling

**Figure 11:**
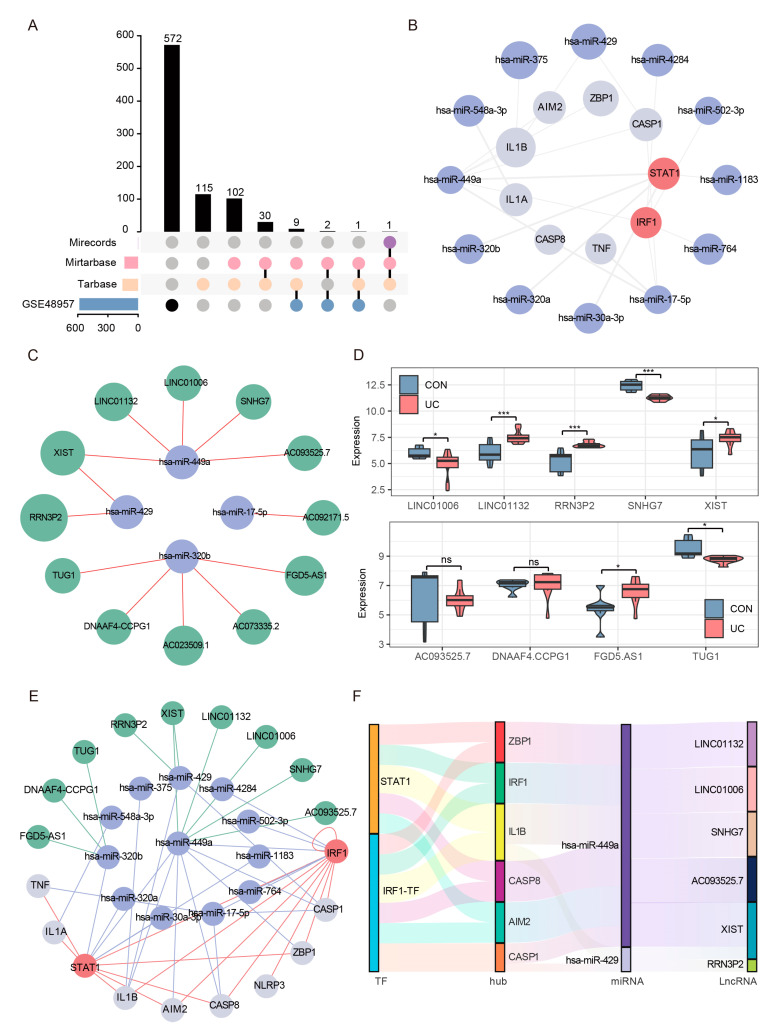
Reliable CeRNA Network Construction. (**A**) The upset plot shows the intersection of the experimentally validated predicted miRNAs in the three databases and the downregulated miRNAs in the validation set GSE48957. (**B**) The miRNA–mRNA network included 7 hub genes, 2 TFs, and 12 regulatory miRNAs. The light gray nodes represent hub genes, red nodes represent TFs, light blue nodes represent miRNAs. (**C**) A total of 4 out of the 12 verified miRNAs predicted 12 experimentally verified (AGO-CLIP > 1 and degradome-seq > 1) lncRNA in the ENCORI database. (**D**) Expression analysis of predicted lncRNAs in data GSE77013, 9 of 12 lncRNAs could be detected. * FDR < 0.05, *** FDR < 0.001, NS, no significance. (**E**) The reliable lncRNA–miRNA–mRNA network was constructed, including 8 hub genes, 2 TFs, 12 miRNAs, and 9 lncRNAs. The light gray nodes represent hub genes, red nodes represent TFs, light blue nodes represent miRNA, and green nodes represents lncRNAs. (**F**) Sankey diagram of final ceRNA network. The squareness represents lncRNAs, miRNAs, and mRNAs, and the size indicates their degree of connection.

The competing endogenous RNA (ceRNA) hypothesis proposes a novel regulatory mechanism, wherein microRNAs (miRNAs) not only exert their effects by targeting messenger RNA (mRNA), but also compete with long non-coding RNAs (lncRNAs) for binding, consequently impacting the expression levels of mRNA. Based on the significant changes and importance of hub genes and TFs in UC, we tried to construct a ceRNA network including multiple mRNA, miRNA, and lncRNA nodes to reveal the molecular regulation mechanism of UC PANoptosis. We first obtained 260 experimentally validated regulatory miRNAs of hub genes and TFs through multiMiR prediction (Supplementary Table S5). We then displayed the expression of predicted miRNAs in the set GSE48957, retained all downregulated miRNAs (Foldchange ≥ 1.5, FDR < 0.05) (Figure 11A), and constructed a credible hub gene–TFs–miRNA network (Figure 11B). Subsequently, 12 experimentally verified lncRNAs interacting with credible miRNAs in the ENCORI database were predicted (Figure 11C). A total of 9 of 12 lncRNAs detectable in the colon were retained through the set GSE77013 screening (Figure 11D). Finally, we successfully constructed a credible ceRNA network, including 10 mRNAs, 12 miRNAs, and 9 lncRNAs (Figure 11E). Through further network topology analysis, we found a sub-network centered on hsa-miR-449a (Figure 11F), which mediated the crosstalk between two TFs (STAT1, IRF1), two sensors (ZBP1, AIM2), two effectors (CASP1/8), and five lncRNAs, suggesting that this network is the core network regulating UC PANoptosis signaling.

### 2.10. Animal Models Validate the Core Genes of PANoptosis Signaling

**Figure 12:**
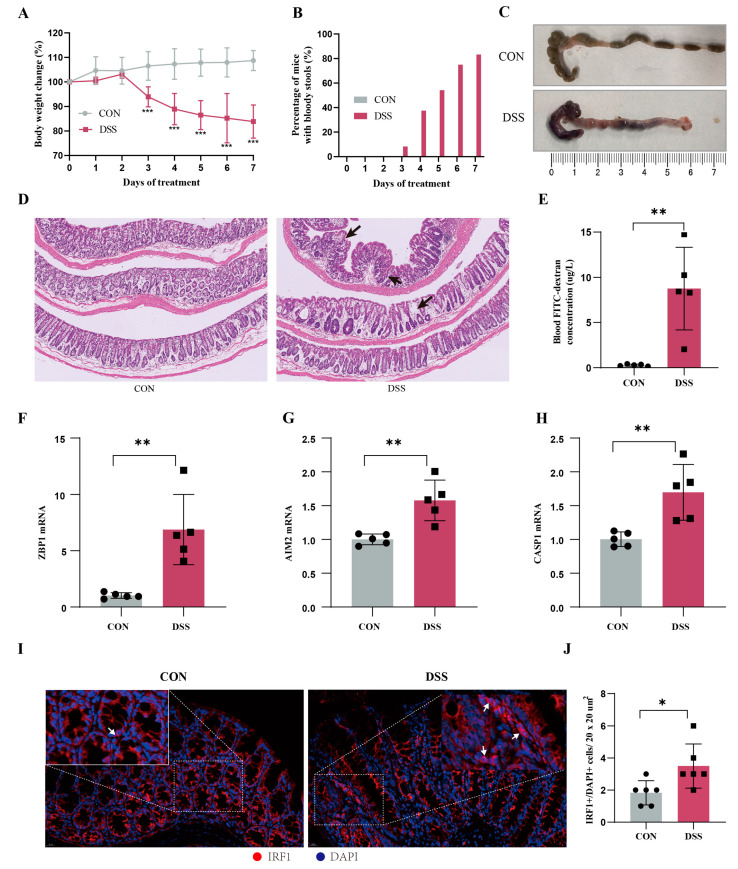
Animal models validate the core genes of PANoptosis signaling. (**A**) Body weight changes (*n* = 6), (**B**) percentage of mice with bloody stools (*n* = 6), and (**C**) colonic length changes (*n* = 6) between CON and UC mice. (**D**) Representative images of HE staining in the colon tissues (magnification ×100) and arrows represents inflammation. (**E**) Blood FITC–dextran concentration (*n* = 5). (**F**–**H**) The mRNA expression levels of ZBP1 (**F**), AIM2 (**G**), and CASP1 (**H**) in UC and CON samples by RT-qPCR (*n* = 5). (**I**) Representative immunofluorescence image of IRF1 between DSS mice and CON mice. Red, IRF1; blue, DAPI nuclear staining. Scale bar, 20 μm. (**J**) Quantification of IRF1^+^/DAPI cells (*n* = 3/group). Data are show as mean ± SD. * *p* < 0.05, ** *p* < 0.01, *** *p* < 0.001.

To further validate the important role of hub genes in UC, we employed the DSS-induced mouse colitis model. Compared with the CON group, mice in DSS group showed obvious weight loss (Figure 12A), increased number of mice with bloody stools (Figure 12B), and decreased colon length (Figure 12C). The histopathological examination and FITC test showed severe inflammatory infiltrates and increased intestinal mucosal permeability in the DSS group (Figure 12D). Then we detected the expression levels of hub genes using RT-qPCR. Compared with the normal group, the expression levels of ZBP1, AIM2, and CASP1 in the UC group were significantly increased (Figure 12E–G). Additionally, IF confirmed that the critical hub gene and TF IRF1 within the nucleus was significantly elevated in DSS group compared to the normal group (Figure 12H). These results provide cross-species evidence supporting the function and regulation of PANoptosis signaling in UC.

## 3. Discussion

As a newly discovered type of cell death, PANoptosis provides an upstream coordination system for multiple death types, including apoptosis, pyroptosis, and necroptosis, enabling it to activate one or more PCDs pathway [21]. In mouse models, the knockdown of key sensor molecules of PANoptosis has been shown to rescue the death fate of innate immune-induced epithelial cells [14,22]. However, studies on the regulatory mode and molecular mechanism of PANoptosis in UC patients are still lacking.

In this study, we constructed a PANoptosis gene set and revealed significant activation of PANoptosis in UC patients. Comprehensive bioinformatics analysis using RRA, cytoHubba, and MCODE network identification, and cross-validation of multiple data sets revealed ZBP1, AIM2, IRF1, NLRP3, CASP1, CASP8, STAT1, IL1A, IL1B, and TNF. Among these molecules, in addition to IL1A, IL1B, and TNF, which have been widely reported to upregulate inflammatory factors in UC, we emphasize ZBP1, AIM2, NLRP3, and CASP1/8 as components of PANoptosome, especially the critical role of the sensor molecules ZBP1 and AIM2 in inducing PANoptosis signaling.

Z-DNA-binding protein 1 (ZBP1) is a powerful innate immune sensor that plays an essential role as a signaling initiator in innate immune responses and PANoptosis [23]. Structural biology studies have shown that ZBP1 interacts with adapter proteins (such as ASC) and other molecules (such as RIPK1) through the Zα and RHIM domain to form the ZBP1-PANoptosome and execute PANoptosis [13,24]. Co-immunoprecipitation and fluorescent co-labeling experiments confirmed that the ZBP1-PANoptosome complex contains ZBP1, RIPK3, RIPK1, CASP8, CASP6, ASC, and NLRP3 [25,26,27], and participates in NLRP3 inflammasome-dependent pyroptosis, CASP8-mediated apoptosis, and MLKL-driven necroptosis [28]. Even if a specific component, such as NLRP3, is knocked out, ZBP1 can still drive the remaining complex to activate cell death [29]. The abnormal activation of ZBP1 also causes embryonic lethality, intestinal cell death, and skin inflammation [30,31]. Absent in melanoma 2 (AIM2) was initially discovered as an interferon-induced tumor suppressor [32] but was later identified as a cytoplasmic double-stranded DNA (dsDNA) sensor that can assemble inflammasomes with ASC and procaspase-1 [33]. The AIM2 inflammasome initiates the innate immune response by cleaving procaspase-1 and converting IL-1β and IL-18 to their mature forms. The AIM2 inflammasome also promotes pyroptosis by converting Gasdermin-D (GSDMD) to the GSDMD-N fragment [34]. In the host immune response induced by infection, AIM2 can form PANoptosome with ZBP1 and another type of inflammasome Pyrin [35]. Immunoprecipitation showed that the AIM2-PANoposome contained AIM 2, ZBP1, Pyrin, ASC, CASP1, CASP8, RIPK3, RIPK 1, and FADD [35,36]. The analysis of seven independent UC cohorts in this study showed that the previously reported PANoptosome sensors ZBP1 and AIM2, as well as the core components that mediate apoptosis, pyroptosis, and necroptosis, CASP1, CASP8, NLRP3, and MLKL, were all upregulated in the UC group, suggesting that there may be a unique pattern of PANoptosis in the mucosa of UC patients.

Another important finding is that IRF1 is not only the hub gene of PANoptosis, but also the transcription factor with the highest prediction score (NES) of hub genes ZBP1, AIM2, CASP1, CASP8, IL1B, NLRP3, and IRF1 itself. Interferon regulatory factor IRF1 (Interferon Regulatory Factor 1) is a member of the IRF family, which is widely involved in innate and acquired immune responses and cell death processes [37,38]. As the earliest discovered interferon regulator, IRF1 is activated by stimuli such as infection or DNA damage and incorporated into the nucleus, thereby initiating the transcription of interferon-related genes. In addition, IRF1 can also act as its own transcription factor, exerting an autoregulatory effect by binding to its own promoter [39]. Studies have found that IRF1 is a transcription factor of the PANoptosome sensor molecules ZBP1 and NLRP3, and knocking out IRF1 will reduce the expression of PANoptosome core molecules such as NLRP3, CSAP1/3/8, and MLKL in the innate immune response [40]. In a UC mouse model, the knockdown of IRF1 also significantly suppressed the expression of colonic apoptosis, pyroptosis, and necroptosis executioner proteins CASP3/7, GSDMD, and MLKL, inhibiting multi-pathway PCDs of IECs [41]. However, studies also show that IRF1 deficiency may maintain intestinal barrier integrity by limiting TNFα-induced IECs shedding [42]; further investigations are needed to clarify its controversial role in UC.

This study also established a reliable ceRNA network for PANoptosis centered on hsa-miR-449a. The hsa-miR-449a can negatively regulate the expression of ZBP1, AIM2, CASP1, CASP8, IL1B, STAT1 and IRF1, and interact with multiple LncRNAs such as XIST and SNHG7. The competing endogenous RNA (ceRNA) hypothesis proposes an essential post-transcriptional regulatory mechanism involving the interaction between multiple RNA molecules, including mRNA, lncRNA, miRNA, etc. [43]. These RNA transcripts act as competing endogenous RNAs (ceRNAs) or natural microRNA sponges, communicating with and co-regulating each other by competing for and sharing microRNA binding sites [44]. In recent years, ceRNA crosstalk, or the interaction between miRNA and lncRNA, has played a critical role in immune-related diseases such as UC and tumors [45,46,47]. Studies have demonstrated that the expression of miR-449a is significantly reduced in colorectal cancer (CAC) tissues associated with colitis compared to paired adjacent non-cancerous tissues (ANT)[48]. Additionally, during the tumorigenesis process from UC to CAC, the level of miR-449a also exhibits a significant decrease [49]. Reduced expression of miR-449a was also associated with advanced clinical stage and poor histology of CAC [50]. In addition, lncRNA XIST mediates epithelial cell inflammatory response through the NF-κB/NLRP3 inflammasome pathway [45,51]. Taken together, the predicted miR-449a and five key LncRNAs may play an essential role in UC progression by targeting the PANoptosis pathway.

## 4. Methods

### 4.1. Datasets and Sample Selection

We searched GEO databases using the keyword “ulcerative colitis”, the filters criteria were as follows: ① human; ② the dataset had at least five healthy control and five UC samples. Finally, seven mRNA datasets (GSE193677, GSE206285, GSE87466, GSE66407, GSE128682, GSE73661, GSE92415), one miRNA dataset (GSE48957), and one lncRNA dataset (GSE77013) were included [52,53,54,55,56,57] (Table 1).

### 4.2. Identification of Differentially Expressed Genes (DEGs)

GEO2R (https://www.ncbi.nlm.nih.gov/geo/geo2r/, accessed on 1 July 2023) was used to obtain the genes expressed differently between UC samples and healthy control samples from microarray datasets. The Benjamini and Hochberg false discovery rate and t-test methods were applied in the GEO2R tool to calculate the false discovery rate (FDR) and pvalue, respectively. Adjusted *p*-value < 0.05 and a |log_2_ (fold change)| ≥ log_2_ (1.5) were considered to be statistically significant. For datasets of RNA-seq, the raw count files were downloaded and differential expression analysis was performed by DESeq2 package (1.38.0) [58]. Volcano plots of the results were drawn through Hiplot Pro (https://hiplot.com.cn/, accessed on 10 July 2023), a comprehensive web service for biomedical data analysis and visualization.

### 4.3. Robust Rank Aggregation (RRA) Analysis

RRA analysis of the RobustRankAggreg package (1.2.1) was used to integrate gene expression profiles identified from microarray (GSE206285) and RNA-seq (GSE193677) datasets, which can identify genes that are differentially expressed concordantly based on the concordance of the ranks [59]. The RRA expression matrix is sorted according to the scores from small to large for subsequent GSEA analysis.

### 4.4. Biological Function and Pathway Enrichment Analysis

Gene Set Enrichment Analysis (GSEA) was performed by the ClusterProfiler (4.7.1.003) package [60]. Gene Ontology (GO) analysis, Kyoto Encyclopedia of Genes and Genomes (KEGG) pathway enrichment analysis, and Reactome pathway enrichment analysis was performed using the OmicShare tools, a free online platform for data analysis (https://www.omicshare.com/tools, accessed on 10 July 2023). FDR < 0.05 was considered significant.

### 4.5. PANoptosis Gene Sets

The PANoptosis genes were obtained from references [21,61]. All genes in the list first use the “bitr” function in the ClusterProfiler (4.7.1.003) package to perform gene name conversion (Symbol to Gene ID); for symbols that cannot be mapped, manually correct them to common symbol names through NCBI.

### 4.6. Gene Set Variation Analysis (GSVA) Analysis

The GSVA analysis was performed using the GSVA (1.46.0) package [62] in R. The gene sets using in GSVA analysis were derived from the subgenre sets of apoptosis, pyroptosis, and necroptosis in the PANoptosis genes sets. Based on the GSVA score, we conducted the differential analysis for these pathways with the limma (3.54.0) package [63] between CON and UC groups.

### 4.7. PPI Network Analysis

Protein–protein interaction analysis uses a string protein database (https://cn.string-db.org/, accessed on 15 July 2023), a functional protein association network assembling all known and predicted proteins. Multiple symbols of DEGs were input with one name per line. PPI network interactions file with medium confidence scores ≥ 0.4 was downloaded. Cytoscape software (version 3.9.1) was used to construct and visualize the networks. Molecular complex detection (MCODE) and cytoHubba plugins with default parameters were used to explore important gene clusters and hub genes [64,65]. The iRegulon (Version: 1.3) plugin was used to screen key transcription factors (TFs) with default parameters [66].

### 4.8. Evaluation of Tissue Infiltrating Immune Cells

Immune cell infiltration analysis was carried out using the CIBERSORT (0.1.0) package [67], which can predict the immune cell composition of tissues using the Cibersort deconvolution algorithm based on input gene expression profiles and the built-in reference set LM22. Permutation (PERM) was established to 1000 for more stable results.

### 4.9. Correction Analysis

The Hmisc (5.1-0) package was used to complete the correlation analysis between genes, and the correlation analysis between genes and immune cells, genes, and immunostimulatory factors was conducted by Hiplot Pro. The immunostimulatory factors were downloaded from the organized gene list of “Immunomodulator” of TISIDB (http://cis.hku.hk/TISIDB/index.php, accessed on 6 March 2023). Additionally, all correlation heatmap visualizations are done with Hiplot Pro.

### 4.10. Small Molecule Agents Screening and Molecular Docking Analysis

The connectivity map database (CMap, https://clue.io/, accessed on 7 August 2023) is a differential gene expression-based drug prediction database, which is primarily used to explore the functional relationships among genes, small molecule compounds, and diseases. The primary protein structures of the target genes were downloaded from The Protein Data Bank database (http://www.rcsb.org, PDB, accessed on 10 August 2023). Home for Researchers (www.home-for-researchers.com, accessed on 10 August 2023) was used to molecularly dock the key targets to small molecule compounds.

### 4.11. CeRNA Network Construction

The multiMiR (1.20.0) package was used to predict hub gene and TF miRNAs, which compiled nearly 50 million records in humans and mice from 14 databases [68]. Choose all miRNAs experimentally validated from the list of predicted records. ENCORI (https://rnasysu.com/encori/, accessed on 10 December 2023) was used to predict miRNA–lncRNA interactions with screening conditions AGO-CLIP and degradome-seq both at >1. The interaction networks were constructed and visualized using Cytoscape, and OmicShare drew a Sankey diagram.

### 4.12. Animal Model of Colitis

Male C57BL/6J mice (~25g) were obtained from Gempharmatech (Chengdu, China). This study was performed in accordance with the recommendations in the Guide for the Care and Use of laboratory Animals of the National Institutes of Health. The protocol was approved by the Committee on the Ethics of Animal Experiments of Chengdu University of Traditional Chinese medicine. Mice were randomized into the control group and DSS group. Mice in DSS group were provided with 2.5% DSS (MP Biomedicals, Shanghai, China) in theit drinking water for 7 days, and the control group was only administered distilled water. The body weight, stool consistency, and fecal bleeding were evaluated daily.

### 4.13. Intestinal Permeability Assay to FITC-Dextran

Intestinal barrier integrity was assessed by permeability to FITC-dextran (Sigma-Aldrich, St. Louis, MO, USA). The experiment was carried out on the last day of exposure to DSS. Before the start of the experiment, the mice fasted for 4 h and were allowed to drink freely. FITC-dextran was orally administered to mice at a weight of 600 mg/kg, and then the mice were returned to the cage, without food but with drinking water. After 2 h of oral administration of FITC-dextran, the mice were anesthetized and blood was collected into heparinized, light-protected tubes and centrifuged (10 min, 12,000× *g*, 4 °C). The concentration of FITC-dextran was analyzed using a fluorescence spectrophotometer (TECAN, Infinite M200, Shanghai, China) and I-Control 2.0 software at the excitation wavelength of 485 nm and the emission wavelength of 528 nm.

### 4.14. H&E and IF Staining

Entire colons were excised postmortem. Colon tissues were fixed with 4% paraformaldehyde (PFA) overnight and were then embedded in paraffin. Colonic sections of 5 mm were obtained and laid flat on a glass slide for H&E or IF staining. For IF staining, the tissue slices were permeabilized with 0.1% Triton X-100 for 15 min, blocked with 1% BSA for 1 h at 37 °C, and then incubated with antibodies against IRF1 (Proteintech, 11335-1-AP, 1:200) at 4 °C overnight. The next day, tissue slices were incubated with Cy3-conjugated Affinipure Goat Anti-Rabbit IgG (SA00009-2, 1:100, Proteintech, Wuhan, China) at 37 °C for 2 h. Then, the sections were rinsed three more times and stained for 5 min with DAPI (Biyuntian, Shanghai, China). The results were imaged by Pannoramic 250FLASH (3DHISTECH, Budapest, Hungary).

### 4.15. RNA Extraction and RT-PCR

RNA was extracted from the intestinal tissues using MolPure^®^ TRIeasy Plus Total RNA Kit (Yeasen Biotechnology, Shanghai, China, 19211ES60) and reverse-transcribed to cDNA using the Hifair^®^ III 1st Strand cDNA Synthesis Kit (Yeasen Biotechnology, 11141ES60). Primer sequences were obtained from PrimerBank (https://pga.mgh.harvard.edu/primerbank/, accessed 20 August 2023) and synthesized at Sangon Biotech (Chengdu, China) (Supplementary Table S1). RT-PCR was performed using the Hieff^®^ qPCR SYBR Green Master Mix (No Rox, Yeasen Biotechnology, 11201ES03) on an QuantGene 9600 (Bioer Technology, Hangzhou, China). PCR amplification was conducted in triplicate for each sample, and the expression of target genes was normalized to β-actin. Relative expression was determined using the 2^−ΔΔCt^ method.

### 4.16. Statistical Analysis

All data analyses were conducted with GraphPad Prism V8.0 software. Data are presented as mean ± SD. Student’s *t* tests were applied for comparisons between two groups, repeatedly measured data were analyzed by repeated measurement analysis of variance. All statistical analyses were two-sided, and *p* < 0.05 was considered to be significant.

## 5. Conclusions

In summary, we confirmed the significant activation of PANoptosis in the UC mucosa from the transcriptome level, determined the high expression of PANoptosome components ZBP1, AIM2, NLRP3, and CASP1/8 in UC, and found that these proteins are associated with inflammatory infiltration of the mucosa and response to biologics.

**Figure 13:**
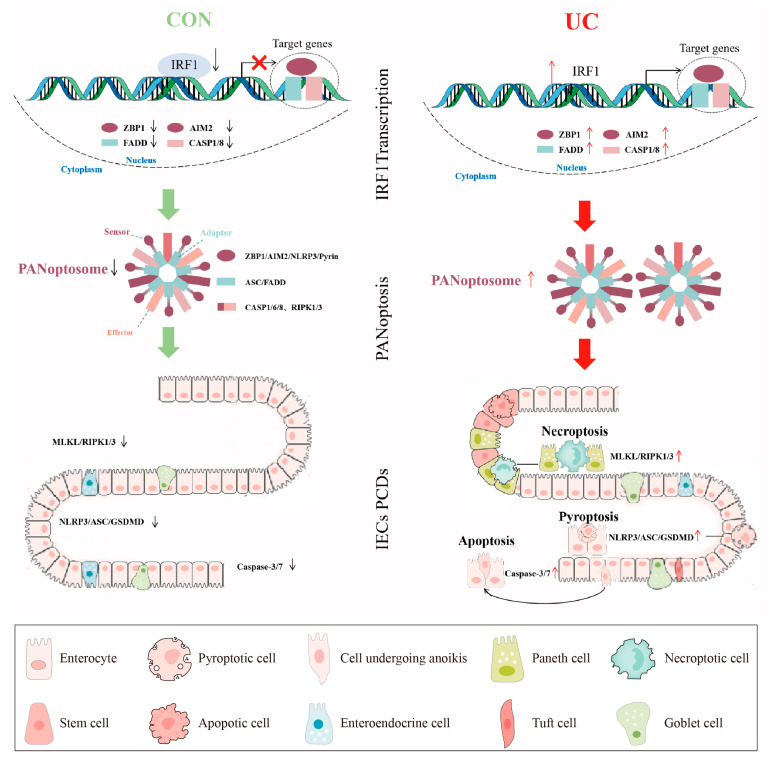
A pictorial summary of IRF1 activating PANoptosis in UC. The green and red arrows represent the activation and inhibition of downstream signals respectively. The red "×" indicates the inhibition of gene transcription expression.

We also built a reliable PANoptosis ceRNA network containing the PANoptosome core molecules, key TFs, and miRNA and lncRNA in the UC mucosa. Based on the above results, we hypothesize that IRF1 as a TF promotes PANoptosome multicomponent expression, activates PANoptosis, and then induces IECs PCDs (Figure 13). These results will help increase the understanding of the relationship between mucosa immune response and colon IECs “Death Landscape” and find a new therapy for inducing and maintaining mucosa healing.

## Figures and Tables

**Table 1:** The information of all the datasets in the study.

GEO ID	RNA Type	Platform	Tissues	Attribute
GSE193677	mRNA	GPL16791	Colonic mucosal	Training set
GSE206285	mRNA	GPL13158	Colonic mucosal	Training set
GSE87466	mRNA	GPL13158	Colonic mucosal	Validation set
GSE66407	mRNA	GPL19833	Colonic mucosal	Validation set
GSE128682	mRNA	GPL21697	Colonic mucosal	Validation set
GSE73661	mRNA	GPL6244	Colonic mucosal	Validation set
GSE92415	mRNA	GPL13158	Colonic mucosal	Validation set
GSE48957	miRNA	GPL14613	Colonic mucosal	Validation set
GSE77013	lncRNA	GPL16956	Colonic mucosal	Validation set

## Data Availability

The datasets used in this paper are available online, as described in the Section 4.

## Supplementary Materials

The following supporting information can be downloaded at: https://www.mdpi.com/article/10.3390/ijms25010348/s1.

## Abbreviations

IECs	Intestinal epithelial cells
UC	Ulcerative colitis
RRA	Robust Rank Aggregation
GSEA	Gene set enrichment analysis
GSVA	Geneset variation analysis
TFs	Transcription factors
CeRNA	Competitive endogenous RNA
PCD	Programmed cell death
FDR	False discovery rate
GO	Gene Ontology
KEGG	Kyoto Encyclopedia of Genes and Genomes
CMap	Connectivity map database
ZBP1	Z-DNA-binding protein 1
AIM2	Absent in melanoma 2
GSDMD	Gasdermin-D
IRF1	Interferon Regulatory Factor 1
CAC	Colorectal cancer

## References

[1] Patankar J.V., Becker C. (2020). Cell death in the gut epithelium and implications for chronic inflammation. Nat. Rev. Gastroenterol. Hepatol..

[2] Subramanian S., Geng H., Tan X.-D. (2020). Cell death of intestinal epithelial cells in intestinal diseases. Sheng Li Xue Bao.

[3] Ungaro R., Mehandru S., Allen P.B., Peyrin-Biroulet L., Colombel J.-F. (2017). Ulcerative colitis. Lancet Lond. Engl..

[4] Guenin-Mace L., Konieczny P., Naik S. (2023). Immune-Epithelial Cross Talk in Regeneration and Repair. Annu. Rev. Immunol..

[5] Bedoui S., Herold M.J., Strasser A. (2020). Emerging connectivity of programmed cell death pathways and its physiological implications. Nat. Rev. Mol. Cell Biol..

[6] Christgen S., Tweedell R.E., Kanneganti T.-D. (2022). Programming inflammatory cell death for therapy. Pharmacol. Ther..

[7] Bulek K., Zhao J., Liao Y., Rana N., Corridoni D., Antanaviciute A., Chen X., Wang H., Qian W., Miller-Little W.A. (2020). Epithelial-derived gasdermin D mediates nonlytic IL-1β release during experimental colitis. J. Clin. Investig..

[8] Schwarzer R., Jiao H., Wachsmuth L., Tresch A., Pasparakis M. (2020). FADD and Caspase-8 Regulate Gut Homeostasis and Inflammation by Controlling MLKL- and GSDMD-Mediated Death of Intestinal Epithelial Cells. Immunity.

[9] Günther C., Martini E., Wittkopf N., Amann K., Weigmann B., Neumann H., Waldner M.J., Hedrick S.M., Tenzer S., Neurath M.F. (2011). Caspase-8 regulates TNF-α-induced epithelial necroptosis and terminal ileitis. Nature.

[10] Bertheloot D., Latz E., Franklin B.S. (2021). Necroptosis, pyroptosis and apoptosis: An intricate game of cell death. Cell Mol. Immunol..

[11] Nagata S., Tanaka M. (2017). Programmed cell death and the immune system. Nat. Rev. Immunol..

[12] Place D.E., Kanneganti T.-D. (2020). The innate immune system and cell death in autoinflammatory and autoimmune disease. Curr. Opin. Immunol..

[13] Karki R., Kanneganti T.-D. (2023). PANoptosome signaling and therapeutic implications in infection: Central role for ZBP1 to activate the inflammasome and PANoptosis. Curr. Opin. Immunol..

[14] Karki R., Sharma B.R., Tuladhar S., Williams E.P., Zalduondo L., Samir P., Zheng M., Sundaram B., Banoth B., Malireddi R.K.S. (2021). Synergism of TNF-α and IFN-γ Triggers Inflammatory Cell Death, Tissue Damage, and Mortality in SARS-CoV-2 Infection and Cytokine Shock Syndromes. Cell.

[15] Wang Y., Kanneganti T.-D. (2021). From pyroptosis, apoptosis and necroptosis to PANoptosis: A mechanistic compendium of programmed cell death pathways. Comput. Struct. Biotechnol. J..

[16] Zhu P., Ke Z.-R., Chen J.-X., Li S.-J., Ma T.-L., Fan X.-L. (2023). Advances in Mechanism and Regulation of PANoptosis: Prospects in Disease Treatment. Front. Immunol..

[17] Innate Immune Inflammatory Cell Death: PANoptosis and PANoptosomes in Host Defense and Disease—PubMed. https://pubmed.ncbi.nlm.nih.gov/36782083/.

[18] NLRP12-PANoptosome activates PANoptosis and Pathology in Response to Heme and PAMPs—PubMed. https://pubmed.ncbi.nlm.nih.gov/37267949/.

[19] Therapeutic Potential of PANoptosis: Innate Sensors, Inflammasomes, and RIPKs in PANoptosomes—PubMed. https://pubmed.ncbi.nlm.nih.gov/37977994/.

[20] Place D.E., Kanneganti T.-D. (2019). Cell death-mediated cytokine release and its therapeutic implications. J. Exp. Med..

[21] Gullett J.M., Tweedell R.E., Kanneganti T.-D. (2022). It’s All in the PAN: Crosstalk, Plasticity, Redundancies, Switches, and Interconnectedness Encompassed by PANoptosis Underlying the Totality of Cell Death-Associated Biological Effects. Cells.

[22] Sharma B.R., Kanneganti T.-D. (2023). Inflammasome signaling in colorectal cancer. Transl. Res. J. Lab. Clin. Med..

[23] Chen X.-Y., Dai Y.-H., Wan X.-X., Hu X.-M., Zhao W.-J., Ban X.-X., Wan H., Huang K., Zhang Q., Xiong K. (2022). ZBP1-Mediated Necroptosis: Mechanisms and Therapeutic Implications. Molecules.

[24] Zheng M., Karki R., Vogel P., Kanneganti T.-D. (2020). Caspase-6 Is a Key Regulator of Innate Immunity, Inflammasome Activation, and Host Defense. Cell.

[25] Malireddi R.K.S., Gurung P., Kesavardhana S., Samir P., Burton A., Mummareddy H., Vogel P., Pelletier S., Burgula S., Kanneganti T.-D. (2020). Innate immune priming in the absence of TAK1 drives RIPK1 kinase activity-independent pyroptosis, apoptosis, necroptosis, and inflammatory disease. J. Exp. Med..

[26] Zhang T., Yin C., Boyd D.F., Quarato G., Ingram J.P., Shubina M., Ragan K.B., Ishizuka T., Crawford J.C., Tummers B. (2020). Influenza Virus Z-RNAs Induce ZBP1-Mediated Necroptosis. Cell.

[27] Christgen S., Zheng M., Kesavardhana S., Karki R., Malireddi R.K.S., Banoth B., Place D.E., Briard B., Sharma B.R., Tuladhar S. (2020). Identification of the PANoptosome: A Molecular Platform Triggering Pyroptosis, Apoptosis, and Necroptosis (PANoptosis). Front. Cell. Infect. Microbiol..

[28] Samir P., Malireddi R.K.S., Kanneganti T.-D. (2020). The PANoptosome: A Deadly Protein Complex Driving Pyroptosis, Apoptosis, and Necroptosis (PANoptosis). Front. Cell. Infect. Microbiol..

[29] Kuriakose T., Man S.M., Malireddi R.K.S., Karki R., Kesavardhana S., Place D.E., Neale G., Vogel P., Kanneganti T.-D. (2016). ZBP1/DAI is an innate sensor of influenza virus triggering the NLRP3 inflammasome and programmed cell death pathways. Sci. Immunol..

[30] de Reuver R., Verdonck S., Dierick E., Nemegeer J., Hessmann E., Ahmad S., Jans M., Blancke G., Van Nieuwerburgh F., Botzki A. (2022). ADAR1 prevents autoinflammation by suppressing spontaneous ZBP1 activation. Nature.

[31] Hubbard N.W., Ames J.M., Maurano M., Chu L.H., Somfleth K.Y., Gokhale N.S., Werner M., Snyder J.M., Lichauco K., Savan R. (2022). ADAR1 mutation causes ZBP1-dependent immunopathology. Nature.

[32] Chen I.-F., Ou-Yang F., Hung J.-Y., Liu J.-C., Wang H., Wang S.-C., Hou M.-F., Hortobagyi G.N., Hung M.-C. (2006). AIM2 suppresses human breast cancer cell proliferation in vitro and mammary tumor growth in a mouse model. Mol. Cancer Ther..

[33] Wang B., Bhattacharya M., Roy S., Tian Y., Yin Q. (2020). Immunobiology and Structural Biology of AIM2 Inflammasome. Mol. Aspects Med..

[34] Du L., Wang X., Chen S., Guo X. (2022). The AIM2 inflammasome: A novel biomarker and target in cardiovascular disease. Pharmacol. Res..

[35] Lee S., Karki R., Wang Y., Nguyen L.N., Kalathur R.C., Kanneganti T.-D. (2021). AIM2 forms a complex with pyrin and ZBP1 to drive PANoptosis and host defence. Nature.

[36] Malireddi R.K.S., Kesavardhana S., Karki R., Kancharana B., Burton A.R., Kanneganti T.-D. (2020). RIPK1 Distinctly Regulates Yersinia-Induced Inflammatory Cell Death, PANoptosis. ImmunoHorizons.

[37] Feng H., Zhang Y.-B., Gui J.-F., Lemon S.M., Yamane D. (2021). Interferon regulatory factor 1 (IRF1) and anti-pathogen innate immune responses. PLoS Pathog..

[38] Tamura T., Ishihara M., Lamphier M.S., Tanaka N., Oishi I., Aizawa S., Matsuyama T., Mak T.W., Taki S., Taniguchi T. (1995). An IRF-1-dependent pathway of DNA damage-induced apoptosis in mitogen-activated T lymphocytes. Nature.

[39] Zhou H., Tang Y.-D., Zheng C. (2022). Revisiting IRF1-mediated antiviral innate immunity. Cytokine Growth Factor Rev..

[40] Kuriakose T., Zheng M., Neale G., Kanneganti T.-D. (2018). IRF1 Is a Transcriptional Regulator of ZBP1 Promoting NLRP3 Inflammasome Activation and Cell Death during Influenza Virus Infection. J. Immunol..

[41] Karki R., Sharma B.R., Lee E., Banoth B., Malireddi R.S., Samir P., Tuladhar S., Mummareddy H., Burton A.R., Vogel P. (2020). Interferon regulatory factor 1 regulates PANoptosis to prevent colorectal cancer. J. Clin. Investig..

[42] Tan G., Huang C., Chen J., Chen B., Shi Y., Zhi F. (2022). An IRF1-dependent Pathway of TNFα-induced Shedding in Intestinal Epithelial Cells. J. Crohn’s Colitis.

[43] Tay Y., Rinn J., Pandolfi P.P. (2014). The multilayered complexity of ceRNA crosstalk and competition. Nature.

[44] Thomson D.W., Dinger M.E. (2016). Endogenous microRNA sponges: Evidence and controversy. Nat. Rev. Genet..

[45] Xu M., Kong Y., Chen N., Peng W., Zi R., Jiang M., Zhu J., Wang Y., Yue J., Lv J. (2022). Identification of Immune-Related Gene Signature and Prediction of CeRNA Network in Active Ulcerative Colitis. Front. Immunol..

[46] Sun C.-C., Zhu W., Li S.-J., Hu W., Zhang J., Zhuo Y., Zhang H., Wang J., Zhang Y., Huang S.X. (2020). FOXC1-mediated LINC00301 facilitates tumor progression and triggers an immune-suppressing microenvironment in non-small cell lung cancer by regulating the HIF1α pathway. Genome Med..

[47] Boutry C., Hastie A., Diez-Domingo J., Tinoco J.C., Yu C.J., Andrews C., Beytout J., Caso C., Cheng H.S., Cheong H.J. (2022). The Adjuvanted Recombinant Zoster Vaccine Confers Long-Term Protection Against Herpes Zoster: Interim Results of an Extension Study of the Pivotal Phase 3 Clinical Trials ZOE-50 and ZOE-70. Clin. Infect. Dis. Off. Publ. Infect. Dis. Soc. Am..

[48] Gu Y., Zhao H., Zheng L., Zhou C., Han Y., Wu A., Jia Z., Xia T., Zhi Q. (2023). Non-coding RNAs and colitis-associated cancer: Mechanisms and clinical applications. Clin. Transl. Med..

[49] Lan S.-H., Lin S.-C., Wang W.-C., Yang Y.-C., Lee J.-C., Lin P.-W., Chu M.-L., Lan K.-Y., Zuchini R., Liu H.-S. (2021). Autophagy Upregulates miR-449a Expression to Suppress Progression of Colorectal Cancer. Front. Oncol..

[50] Feng Y., Dong Y.-W., Song Y.-N., Xiao J.-H., Guo X.-Y., Jiang W.-L., Lu L.-G. (2018). MicroRNA-449a is a potential predictor of colitis-associated colorectal cancer progression. Oncol. Rep..

[51] Ma M., Pei Y., Wang X., Feng J., Zhang Y., Gao M.-Q. (2019). LncRNA XIST mediates bovine mammary epithelial cell inflammatory response via NF-κB/NLRP3 inflammasome pathway. Cell Prolif..

[52] Argmann C., Hou R., Ungaro R.C., Irizar H., Al-Taie Z., Huang R., Kosoy R., Venkat S., Song W.M., Di’Narzo A.F. (2023). Biopsy and blood-based molecular biomarker of inflammation in IBD. Gut.

[53] Pavlidis P., Tsakmaki A., Pantazi E., Li K., Cozzetto D., Bell J.D., Yang F., Lo J.W., Alberts E., Sa A.C.C. (2022). Interleukin-22 regulates neutrophil recruitment in ulcerative colitis and is associated with resistance to ustekinumab therapy. Nat. Commun..

[54] Li K., Strauss R., Ouahed J., Chan D., Telesco S.E., Shouval D.S., Canavan J.B., Brodmerkel C., Snapper S.B., Friedman J.R. (2018). Molecular Comparison of Adult and Pediatric Ulcerative Colitis Indicates Broad Similarity of Molecular Pathways in Disease Tissue. J. Pediatr. Gastroenterol. Nutr..

[55] Sandborn W.J., Feagan B.G., Marano C., Zhang H., Strauss R., Johanns J., Adedokun O.J., Guzzo C., Colombel J.-F., Reinisch W. (2014). Subcutaneous golimumab induces clinical response and remission in patients with moderate-to-severe ulcerative colitis. Gastroenterology.

[56] Van der Goten J., Vanhove W., Lemaire K., Van Lommel L., Machiels K., Wollants W.-J., De Preter V., De Hertogh G., Ferrante M., Van Assche G. (2014). Integrated miRNA and mRNA expression profiling in inflamed colon of patients with ulcerative colitis. PLoS ONE.

[57] Padua D., Mahurkar-Joshi S., Law I.K.M., Polytarchou C., Vu J.P., Pisegna J.R., Shih D., Iliopoulos D., Pothoulakis C. (2016). A long noncoding RNA signature for ulcerative colitis identifies IFNG-AS1 as an enhancer of inflammation. Am. J. Physiol. Gastrointest. Liver Physiol..

[58] Love M.I., Huber W., Anders S. (2014). Moderated estimation of fold change and dispersion for RNA-seq data with DESeq2. Genome Biol..

[59] Kolde R., Laur S., Adler P., Vilo J. (2012). Robust rank aggregation for gene list integration and meta-analysis. Bioinforma Oxf. Engl..

[60] Wu T., Hu E., Xu S., Chen M., Guo P., Dai Z., Feng T., Zhou L., Tang W., Zhan L. (2021). clusterProfiler 4.0: A universal enrichment tool for interpreting omics data. Innov. Camb. Mass..

[61] Pan H., Pan J., Li P., Gao J. (2022). Characterization of PANoptosis patterns predicts survival and immunotherapy response in gastric cancer. Clin. Immunol..

[62] Hänzelmann S., Castelo R., Guinney J. (2013). GSVA: Gene set variation analysis for microarray and RNA-seq data. BMC Bioinform..

[63] Ritchie M.E., Phipson B., Wu D., Hu Y., Law C.W., Shi W., Smyth G.K. (2015). limma powers differential expression analyses for RNA-sequencing and microarray studies. Nucleic Acids Res..

[64] Lin C.-Y., Chin C.-H., Wu H.-H., Chen S.-H., Ho C.-W., Ko M.-T. (2008). Hubba: Hub objects analyzer–A framework of interactome hubs identification for network biology. Nucleic Acids Res..

[65] Chin C.-H., Chen S.-H., Wu H.-H., Ho C.-W., Ko M.-T., Lin C.-Y. (2014). cytoHubba: Identifying hub objects and sub-networks from complex interactome. BMC Syst. Biol..

[66] Janky R., Verfaillie A., Imrichová H., Van de Sande B., Standaert L., Christiaens V., Hulselmans G., Herten K., Sanchez M.N., Potier D. (2014). iRegulon: From a gene list to a gene regulatory network using large motif and track collections. PLoS Comput. Biol..

[67] Newman A.M., Liu C.L., Green M.R., Gentles A.J., Feng W., Xu Y., Hoang C.D., Diehn M., Alizadeh A.A. (2015). Robust enumeration of cell subsets from tissue expression profiles. Nat. Methods.

[68] Ru Y., Kechris K.J., Tabakoff B., Hoffman P., Radcliffe R.A., Bowler R., Mahaffey S., Rossi S., Calin G.A., Bemis L. (2014). The multiMiR R package and database: Integration of microRNA-target interactions along with their disease and drug associations. Nucleic Acids Res..

